# Role of non-macrophage cell-derived HMGB1 in oxaliplatin-induced peripheral neuropathy and its prevention by the thrombin/thrombomodulin system in rodents: negative impact of anticoagulants

**DOI:** 10.1186/s12974-019-1581-6

**Published:** 2019-10-30

**Authors:** Maho Tsubota, Ryotaro Fukuda, Yusuke Hayashi, Takaya Miyazaki, Shin Ueda, Rika Yamashita, Nene Koike, Fumiko Sekiguchi, Hidenori Wake, Shuji Wakatsuki, Yuka Ujiie, Toshiyuki Araki, Masahiro Nishibori, Atsufumi Kawabata

**Affiliations:** 1 Laboratory of Pharmacology and Pathophysiology, Faculty of Pharmacy, Kindai University (formerly known as Kinki University), 3-4-1 Kowakae, Higashi-osaka, 577-8502 Japan; 2 Department of Pharmacology, Okayama University Graduate School of Medicine, Okayama, 700-8558 Japan; 3 Department of Peripheral Nervous System Research, National Institute of Neuroscience, National Center of Neurology and Psychiatry, Kodaira, Tokyo 187-8502 Japan

**Keywords:** Oxaliplatin, Chemotherapy-induced peripheral neuropathy (CIPN), High mobility group box 1 (HMGB1), Thrombomodulin, Thrombin

## Abstract

**Background:**

Macrophage-derived high mobility group box 1 (HMGB1), a damage-associated molecular pattern (DAMP) protein, plays a key role in the development of chemotherapy-induced peripheral neuropathy (CIPN) caused by paclitaxel in rodents. Endothelial thrombomodulin (TM) promotes thrombin-induced degradation of HMGB1, and TMα, a recombinant human soluble TM, abolishes peripheral HMGB1-induced allodynia in mice. We thus examined whether HMGB1, particularly derived from macrophages, contributes to oxaliplatin-induced neuropathy in mice and analyzed the anti-neuropathic activity of the TM/thrombin system.

**Methods:**

CIPN models were created by the administration of oxaliplatin in mice and rats, and the nociceptive threshold was assessed by von Frey test or paw pressure test. Macrophage-like RAW264.7 cells were stimulated with oxaliplatin in vitro. Proteins were detected and/or quantified by Western blotting, immunostaining, or enzyme-linked immunosorbent assay.

**Results:**

Intraperitoneal administration of an anti-HMGB1-neutralizing antibody (AB) at 1 mg/kg prevented the oxaliplatin-induced allodynia in mice and rats. Antagonists of Toll-like receptor (TLR) 4, receptor for advanced glycation end products (RAGE) and CXCR4 among the HMGB1-targeted pro-nociceptive receptors, also mimicked the anti-neuropathic activity of AB in mice. Macrophage accumulation in the sciatic nerve was observed in mice treated with paclitaxel, but not oxaliplatin, and neither macrophage depletion nor inhibitors of macrophage activation affected oxaliplatin-induced allodynia. Oxaliplatin was 10- to 100-fold less potent than paclitaxel in releasing HMGB1 from macrophage-like RAW264.7 cells. Like AB, TMα at 10 mg/kg prevented the oxaliplatin-induced allodynia in mice as well as rats, an effect abolished by argatroban at 10 mg/kg, a thrombin inhibitor. The anti-neuropathic activity of TMα in oxaliplatin-treated mice was suppressed by oral anticoagulants such as warfarin at 1 mg/kg, dabigatran at 75 mg/kg, and rivaroxaban at 10 mg/kg, but not antiplatelet agents such as aspirin at 50 mg/kg and clopidogrel at 10 mg/kg. Repeated administration of the anticoagulants gradually developed neuropathic allodynia and elevated plasma HMGB1 levels in mice treated with a subeffective dose of oxaliplatin.

**Conclusions:**

Our data thus suggests a causative role of HMGB1 derived from non-macrophage cells in oxaliplatin-induced peripheral neuropathy and a thrombin-dependent anti-neuropathic activity of exogenous TMα and, most probably, endogenous TM.

## Background

Chemotherapy-induced peripheral neuropathy (CIPN), one of the most serious side effects of anti-cancer agents, is a common cause for dose reduction or cessation of cancer treatment. Paclitaxel, oxaliplatin, cisplatin, vincristine, and bortezomib are the most common agents provoking CIPN, although these chemotherapeutics exhibit anti-mitotic activity through different molecular mechanisms [1]. Preclinical studies using animal models of CIPN suggest the involvement of multiple mechanisms, such as mitochondrial dysfunction, oxidative stress, damage-associated molecular patterns (DAMPs), neuroimmune crosstalk, altered function, or expression of ion channels in the pathogenesis of CIPN [2–6].

Emerging evidence has unveiled the role of high mobility group box 1 (HMGB1), a DAMP protein, as a pro-nociceptive mediator in the peripheral tissue and spinal cord [7–10]. HMGB1, a nuclear protein, is passively released from necrotic cells and actively secreted by certain cells including macrophages. Extracellular HMGB1 appears to exist in two distinct active forms, depending on redox states. A fully reduced (all-thiol) form of HMGB1 that has three cysteine residues (C^23^, C^45^, and C^106^) in a thiol form is capable of activating the receptor for advanced glycation end products (RAGE) and accelerating the CXCL12/CXCR4 signaling, while an oxidized (disulfide) form of HMGB1 that has a C^23^-C^45^ intramolecular disulfide bridge strongly drives inflammation through Toll-like receptors (TLRs) 2 and 4 [11, 12]. RAGE and TLR4 mediate the allodynia or hyperalgesia caused by intraplantar or intrathecal administration of HMGB1 in all-thiol and disulfide forms, respectively, in rodents [8, 10]. Recent evidence has also indicated the involvement of TLR5 in pain processing by HMGB1 [13]. Most recently, we have demonstrated that inactivation of macrophage-derived HMGB1 by an anti-HMGB1-neutralizing antibody or a recombinant soluble form of human thrombomodulin (TM), referred to as TMα, completely prevents the development of paclitaxel-induced peripheral neuropathy [4, 6]. Full-length TM, an endothelial transmembrane protein, is composed of five domains, D1–D5, and triggers thrombin-dependent activation of protein C, thereby exhibiting anticoagulant activity [14]. TMα consisting of extracellular D1–D3 retains the potency of TM in activating protein C [14] and is used for the treatment of disseminated intravascular coagulation in Japan. Most interestingly, TMα sequesters HMGB1 with D1 and promotes degradation of HMGB1 by thrombin binding to D2 [14], thereby inhibiting HMGB1-dependent hyperalgesia/allodynia in a thrombin-dependent manner [15, 16].

In the present study, we examined the roles of HMGB1 and macrophages in oxaliplatin-induced peripheral neuropathy in mice and analyzed the effect of TMα alone or in combination with various anticoagulants. Here, we show that, in contrast to the critical role of macrophage-derived HMGB1 in the CIPN caused by paclitaxel [6], oxaliplatin-induced peripheral neuropathy involves HMGB1, but is largely independent of macrophages, and that the TM/thrombin system restrains CIPN most probably by inactivating HMGB1 in the bloodstream.

## Methods

### Animals

Male ddY mice (4–5 weeks old) and male Wistar rats (6–10 weeks old) were purchased from Kiwa Laboratory Animals Co., Ltd. (Wakayama, Japan) and housed in a room maintained at about 24 °C under a 12-h day/night cycle with free access to food and water. Newborn male or female Sprague-Dawley rats (2 days old) were also obtained from Kiwa Laboratory Animals Co., Ltd. for the preparation of primary culture of Schwann cells. The number of animals used is mentioned in the legend for figures. All protocols of animal experiments were approved by the Committee for the Care and Use of Laboratory Animals at Kindai University and were in accordance with the NIH guidelines (Guide for Care and Use of Laboratory Animals, NIH Publication 86-23).

### Major chemicals

The anti-HMGB1-neutralizing rat monoclonal antibody and control IgG were made in house, and the specificity of the antibody is described elsewhere [17]. Recombinant human soluble thrombomodulin [thrombomodulin alfa (TMα), Recomodulin®] was provided by Asahi Kasei Pharma (Tokyo, Japan). Low molecular weight heparin (LMWH; molecular weight, 4500–6500; 79.5 U/mg) was a gift from Fuso Pharmaceutical Industries, Ltd. (Osaka, Japan). Lipopolysaccharide from *Rhodobacter sphaeroides* (LPS-RS) and TAK-242 was purchased from InvivoGen (San Diego, CA, USA) and Cayman Chemical Company (Ann Arbor, MI, USA), respectively. FPS-ZM1 was obtained from Merck Millipore (Burlington, MA, USA). AMD3100, TH1020, ethyl pyruvate, minocycline, argatroban, aspirin, and clopidogrel were purchased from Sigma-Aldrich (St. Louis, MO, USA), and liposomal clodronate (Clophosome-A) and the control liposome were from FormuMax Scientific, Inc. (Sunnyvale, CA, USA). Oxaliplatin and warfarin were provided from FUJIFILM Wako Pure Chemical Corporation (Osaka, Japan), and dabigatran and rivaroxaban were from Combi-Blocks, Inc. (San Diego, CA, USA) and Selleck Chemicals Co., Ltd. (Houston, TX, USA), respectively. Recombinant all-thiol HMGB1 was obtained from HMGBiotech (Milan, Italy). Paclitaxel was purchased from Bristol-Myers Squibb (New York, NY, USA). Oxaliplatin was dissolved in 5% glucose solution, and paclitaxel was in a solution containing 17% Cremophor EL (Nacalai Tesque, Kyoto, Japan), 17% ethanol, and 66% saline. The antibody and non-immune IgG were dissolved in 0.01 M phosphate-buffered saline, and TMα was in saline containing 0.002% Tween 80. FPS-ZM1 was dissolved in saline containing 10% Tween 80 and 0.5% dimethyl sulfoxide (DMSO), and TAK-242 was in saline containing 3.3% DMSO. LMWH, LPS-RS, AMD3100, ethyl pyruvate, minocycline, and argatroban were dissolved in saline. Warfarin was dissolved in distilled water, and dabigatran and rivaroxaban were in 0.5% carboxymethyl cellulose containing 0.4% DMSO and in 0.5% methyl cellulose containing 0.2% Tween 80, respectively. Aspirin and clopidogrel were suspended and dissolved, respectively, in 0.5% carboxymethyl cellulose.

### Creation of mouse and/or rat models for peripheral neuropathy caused by oxaliplatin and paclitaxel

To create a mouse model for oxaliplatin-induced peripheral neuropathy, as described elsewhere [18], oxaliplatin at 5 mg/kg was administered i.p. once to ddY mice, which rapidly developed mechanical allodynia. A subeffective dose, 1 mg/kg, of oxaliplatin was administered i.p. to mice in experiments studying the aggravation of CIPN by anticoagulants. On the other hand, a rat model for oxaliplatin-induced peripheral neuropathy was prepared by repeated administration of oxaliplatin in rats according to the previously reported method [19] with minor modifications, since a single administration of oxaliplatin failed to develop reproducible neuropathic pain in the rats in our preliminary experiments. Briefly, oxaliplatin at 5 mg/kg was repeatedly administered i.p. every third day (days 0, 3, 6, and 9) to Wistar rats, four times in total, which slowly developed mechanical hyperalgesia and allodynia. For comparison, a mouse model for paclitaxel-induced peripheral neuropathy was created by repeated i.p. administration of paclitaxel at 4 mg/kg on days 0, 2, 4, and 6.

### Assessment of mechanical nociceptive threshold in mice and rats

To detect mechanical allodynia, nociceptive threshold in mouse or rat hind paw was measured by von Frey test according to the up-down method [20]. The animals were placed on a risen wire mesh floor and covered a transparent plastic box (mouse 10 × 10 × 10 cm, rat 34.2 × 29.4 × 17.8 cm). After acclimatization, the plantar surface of the right hind paw was stimulated for 6 s with von Frey filaments of distinct strength (Aesthesio®, DanMic Global LCC, San Jose, CA, USA) (0.008, 0.02, 0.04, 0.07, 0.16, 0.4, 0.6, and 1.0 g for mice; 2, 4, 6, 8, 10, 15, 20, 26, and 60 g for rats). The 50% nociceptive threshold to cause escape behavior was calculated as reported previously [20]. In rats, the paw pressure test was also employed to detect mechanical hyperalgesia using an analgesia meter (MK-300, Muromachi Kikai Co., Tokyo, Japan) [9]. Pressure was applied to the rat right hind paw with an increasing rate of 30 g/s. A pressure value to cause escape behavior was detected as the mechanical nociceptive threshold. A cutoff value of 500 g was used in order to prevent damage to the hind paw. The thresholds are shown as the percentage of the average of baseline values.

### Assessment of cold allodynia

After acclimatization in a transparent square container (10 × 10 × 10 cm) for 1 h, cold allodynia in mice was evaluated as described elsewhere [21, 22]. Briefly, the plantar center of the hind paw was exposed to 20 μl of acetone, and the nociceptive responses were scored as follows: 0, no response; 1, quick withdrawal, flick, or stamp of the paw; 2, prolonged withdrawal or repeated flicking of the paw; and 3, repeated flicking of the paw with licking directed at the ventral side of the paw. The acetone challenge was performed alternately three times to each of the right and left hind paws. The data are shown as the total scores in response to six challenges.

### Drug administration schedules

The action mechanisms of the major drugs used in the present study are summarized in Table 1. To examine the preventive effects on the oxaliplatin-induced peripheral neuropathy in mice, the anti-HMGB1-neutralizing rat monoclonal antibody or non-immune IgG at 1 mg/kg; TMα at 1 or 10 mg/kg; LMWH at 2.5 mg/kg, known to block RAGE; FPS-ZM1 at 1 mg/kg, a RAGE antagonist; LPS-RS at 0.5 mg/kg and TAK-242 at 3 mg/kg, TLR4 antagonists; and AMD3100 at 8 mg/kg, a CXCR4 antagonist, were administered i.p. 1 h before i.p. oxaliplatin at 5 mg/kg. In one experiment, TMα at 1 mg/kg was administered three times, 1 h before and 5 h and 2 days after oxaliplatin treatment. In the rat model, the anti-HMGB1 neutralizing antibody or non-immune IgG at 1 mg/kg and TMα at 1 or 3 mg/kg were repeatedly administered i.p. 1 h before and 24 h after each dose of oxaliplatin, eight times in total, i.e., on days 0, 1, 3, 4, 6, 7, 9, and 10 of oxaliplatin treatment. To evaluate the therapeutic effect on the oxaliplatin-induced neuropathic allodynia in mice, the anti-HMGB1-neutralizing antibody or non-immune IgG at 1 mg/kg; TMα at 0.1, 1, or 3 mg/kg; LMWH at 2.5 mg/kg; FPS-ZM1 at 1 mg/kg; LPS-RS at 0.5 mg/kg; TAK-242 at 3 mg/kg; and AMD3100 at 8 mg/kg were administered i.p. 8 days after i.p. oxaliplatin at 5 mg/kg. To test whether macrophages are involved in the oxaliplatin-induced peripheral neuropathy, minocycline at 30 mg/kg was administered i.p. 24 h and 1 h before and daily for six consecutive days after i.p. oxaliplatin at 5 mg/kg, and ethyl pyruvate at 80 mg/kg was administered i.p. 24 h and 1 h before and on days 1, 3, 5, and 7 after oxaliplatin treatment. To check the effects of anticoagulants and antiplatelet agents on the TMα-induced prevention of oxaliplatin-induced neuropathic allodynia, argatroban at 10 mg/kg [23] was administered i.p. to mice 30 min before i.p. TMα at 10 mg/kg, i.e., 1 h before i.p. oxaliplatin at 5 mg/kg; dabigatran at 75 mg/kg [24]; rivaroxaban at 10 mg/kg [25]; aspirin at 50 mg/kg [26]; and clopidogrel at 10 mg/kg [27] were administered orally in the same time schedule. Warfarin at 1 mg/kg [25] and also rivaroxaban at 10 mg/kg were administered orally 1, 24, and 48 h before i.p. oxaliplatin at 5 mg/kg, three times in total, and TMα at 10 mg/kg was administered i.p. 30 min before the oxaliplatin treatment. To test whether anticoagulants promote the oxaliplatin-induced peripheral neuropathy, argatroban at 10 mg/kg and rivaroxaban at 10 mg/kg were administered i.p. and orally, respectively, 1 h before and daily for six consecutive days after a single i.p. administration of oxaliplatin at 1 mg/kg, a subeffective dose, which was confirmed in our preliminary dose-response experiments.

**Table 1:** The mechanism of action of drugs

Drug	Mechanisms
Anti-HMGB1-neutralizing antibody (HMGB1-Ab)	Inactivation of HMGB1
LPS-RS	Blockade of TLR4
Low molecular weight heparin (LMWH)	Blockade of RAGE
AMD3100	Blockade of CXCR4
TAK-242	Blockade of TLR4
FPS-ZM1	Blockade of RAGE
Thrombomodulin α	Inactivation of HMGB1
Liposomal clodronate (Cld)	Depletion of macrophage
Minocycline (Mino)	Inactivation of macrophage/microglia
Ethyl pyruvate (EP)	Inhibition of HMGB1 release from macrophage
Argatroban (AT)	Inhibition of thrombin
Warfarin (War)	Antagonism of vitamin K
Dabigatran (Dabi)	Inhibition of thrombin
Rivaroxaban	Inhibition of Factor Xa
Aspirin (ASA)	Inhibition of COX
Clopidogrel (Clo)	Blockade of P2Y_12_

The mechanism of action of major agents used in the present study

### Macrophage depletion and assessment of macrophage accumulation in the sciatic nerve in mice

To deplete macrophages, liposomal clodronate at 1.05 mg/mouse was administered i.p. twice 24 h before and on day 7 after i.p. oxaliplatin at 5 mg/kg. Successful macrophage depletion in the spleen isolated from the mice sacrificed by cervical dislocation was confirmed by flow cytometry using both anti-CD11b (a marker for monocyte, macrophage, and microglia) and anti-F4/80 (a marker for macrophage and microglia) antibodies, as described previously [28].

Immunofluorescence staining of macrophages in the sciatic nerve was performed on day 8 or 9 after i.p. oxaliplatin at 5 mg/kg or on day 9 after the onset of paclitaxel treatment. Briefly, the mice were anesthetized with i.p. administration of a mixture of midazolam at 4 mg/kg and medetomidine at 0.3 mg/kg, followed by i.p. sodium pentobarbital at 10 mg/kg, and transcardially perfused with ice-cold saline in a volume of 20 ml and then with 4% paraformaldehyde in 0.1 M phosphate buffer in a volume of 50 ml. The excised sciatic nerve was subjected to immunofluorescence staining using a rat anti-mouse F4/80 monoclonal antibody (Bio-rad, Hercules, CA, USA) (1:1000 dilution), Cy3-conjugated goat anti-rat IgG antibody (Thermo Fisher Scientific) (1:400 dilution), and 4,6-diamidino-2-phenylindole dihydrochloride (DAPI) (Sigma-Aldrich) (1: 2000 dilution), as described elsewhere [6]. The F4/80-immunopositive cells were observed by confocal laser fluorescence microscopy (FV10C-O, Olympus, Tokyo, Japan), photographed, and counted in each visual field.

### Determination of plasma HMGB1 levels and tissue protein expression of HMGB1, TLR4, RAGE, and CXCR4 in the DRG, sciatic nerve, and/or hind paw of the mice treated with oxaliplatin

Blood sampling and excision of the DRG, sciatic nerves, and hind paws from mice were performed 5 h and 3 and/or 8 days after administration of oxaliplatin. The blood was also collected from rats 5 h and 3 and 12 days after the onset of oxaliplatin treatment.

In the mice or rats anesthetized as described above (the doses of midazolam and medetomidine were 2 and 0.15 mg/kg, respectively, for rats), the citrated blood was withdrawn from the abdominal aorta. The DRG at the L4 to L6 spinal levels, sciatic nerves, and hind paws were excised from the mice. Plasma HMGB1 levels were measured using an ELISA kit (Fuso Pharmaceutical Industries, Ltd.), immediately after blood collection without freeze-thawing, because thrombin might gradually degrade HMGB1 during plasma thawing [29]. Western blot analysis for the determination of protein levels of HMGB1, TLR4, RAGE, and CXCR4 in tissue samples was conducted, as reported previously [6]. The primary antibodies used were anti-HMGB1 and anti-RAGE rabbit polyclonal antibodies (Abcam, Cambridge, UK) (1:5000 and 1:1000 dilution, respectively), anti-TLR4 rabbit polyclonal antibody (Santa Cruz Biotechnology, Inc., Santa Cruz, CA, USA) (1:10000 dilution), anti-CXCR4 rabbit polyclonal antibody (Novus Biologicals, Littleton, CO, USA) (1:1000 dilution), and anti-glyceraldehyde 3-phosphate dehydrogenase (GAPDH) rabbit polyclonal antibody (Santa Cruz Biotechnology, Inc.) (1:5000 dilution). A horseradish peroxidase (HRP)-conjugated anti-rabbit IgG goat antibody (Cell Signaling Technology, Beverly, MA, USA) (1:5000 dilution) was used as a secondary antibody. The immunoreactive proteins were visualized with a chemiluminescence detection reagent (Chemi-Lumi One Super, Nacalai Tesque, Inc., Kyoto, Japan), and the bands detected by Image Quant 400 (GE Healthcare, Little Chalfont, Buckinghamshire, UK) were quantified using densitometric software (ImageJ downloaded from https://imagej.nih.gov/ij/download.html).

### Immunofluorescence staining of HMGB1 in DRG

The DRG at the L5 spinal level was collected 5 h and 8 days after i.p. oxaliplatin at 5 mg/kg, fixed in 4% paraformaldehyde in 0.1 M phosphate buffer, and subjected to immunofluorescence staining using a chicken anti-HMGB1 polyclonal antibody (#326059669, SHINO-TEST Corporation, Tokyo, Japan) (1:1000 dilution), Alexa Fluor 568-conjugated anti-chicken IgY (H + L) antibody (A-11041, Thermo Fisher Scientific Corporation, Carlsbad, CA, USA) (1:1000 dilution), as mentioned above.

### Measurement of prothrombin time

Prothrombin time was measured in the mice treated with warfarin or rivaroxaban. Warfarin at 1 mg/kg was administered orally 1, 24, and 48 h before blood sampling. Rivaroxaban at 10 mg/kg was administered repeatedly in the same manner or administered once 1 h before blood sampling. The citrated blood was collected, as mentioned above, and the prothrombin time was measured using a kit (ThromboCheck PT Plus®, Sysmex Corporation, Kobe, Japan).

### Measurement of HMGB1 release in mouse macrophage-like RAW264.7 cells and in primary culture of rat Schwann cells

RAW 264.7 cells were cultured in RPMI1640 medium (FUJIFILM Wako Pure Chemical Corporation, Osaka, Japan) containing 10% fetal calf serum (FCS; Nichirei Bioscience, Tokyo, Japan), 100 U/ml penicillin (Nacalai Tesque, Inc.), 100 μg/ml streptomycin (Nacalai Tesque, Inc.) under the conditions of 37 °C, and 5% CO_2_ using a tissue culture dish (100 × 20 mm). Primary cultures of Schwann cells were prepared from 2-day-old Sprague-Dawley rats, as previously described [30]. The collected and purified cells were seeded and cultured in a poly-l-ornithine-coated culture dish filled with a medium containing 10% FCS, 2 μM forskolin, and 20 ng/ml recombinant human heregulin-β1 (Sigma-Aldrich). RAW264.7 cells (36 × 10^4^ cells/well) and rat primary Schwann cells (30 × 10^4^ cells/well) were stimulated with oxaliplatin at 0.1–10 μM or paclitaxel at 0.001–1 μM for 24 or 48 h, and HMGB1 released into the extracellular space was determined by ELISA, as mentioned above.

### Statistics

Data are shown as mean ± SEM In statistical analyses of parametric data, Student’s *t* test was used for comparisons of two-group data, and ANOVA followed by Tukey’s test was employed for comparisons among three groups or more. For non-parametric analyses, Wilcoxon’s *t* test was used for two-group comparisons, and Kruskal-Wallis’s *H* test followed by the LSD test was performed for comparisons of three or more group data. A significant difference was assumed at *P* < 0.05.

## Results

### Involvement of HMGB1 and its target molecules in oxaliplatin-induced peripheral neuropathy in mice and rats

**Fig. 1:**
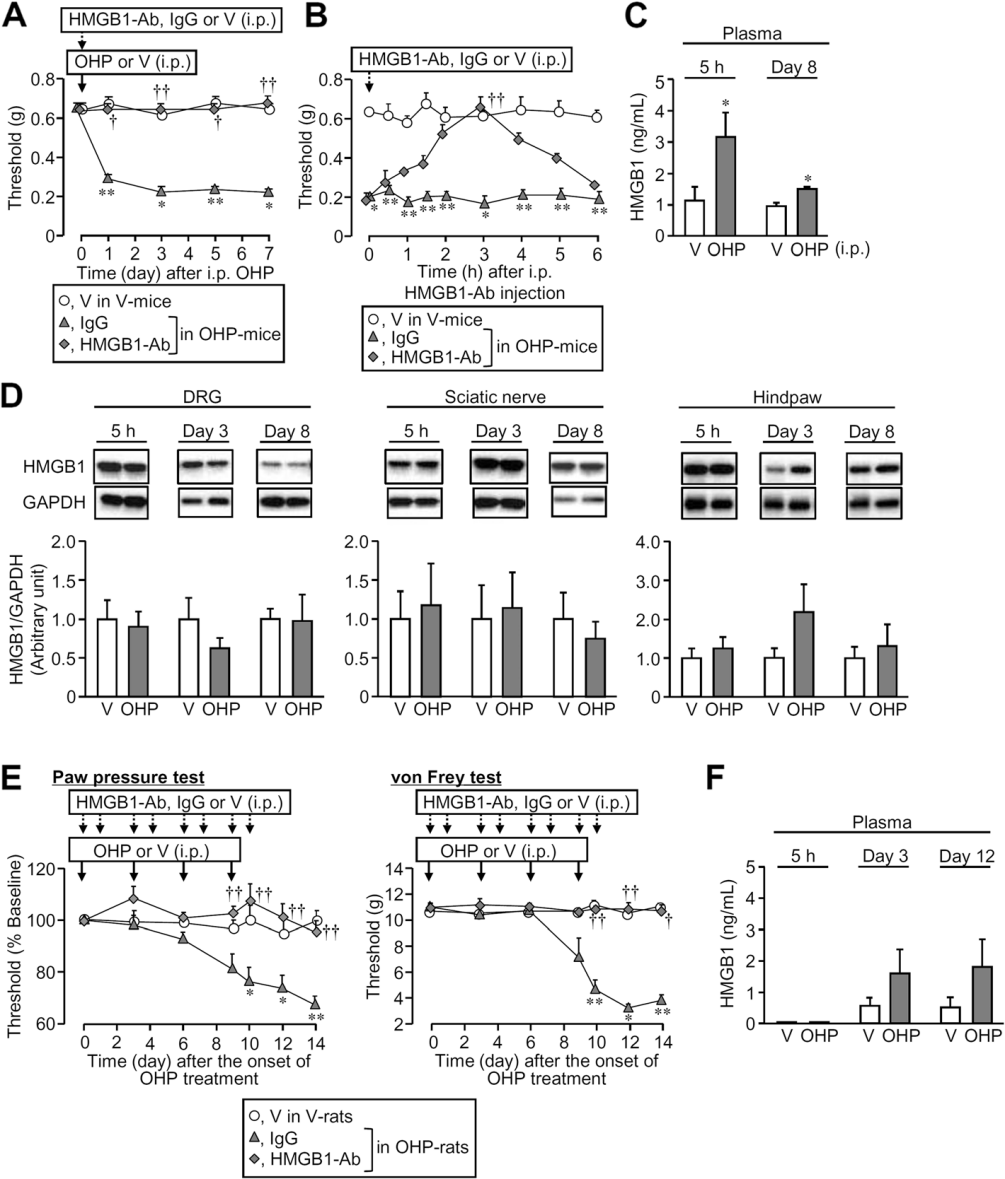
Involvement of endogenous HMGB1 in the oxaliplatin (OHP)-induced neuropathic allodynia in rodents. **a**, **b** Preventive (**a**) and therapeutic (**b**) effects of an anti-HMGB1-neutralizing antibody (HMGB1-Ab) on the OHP-induced mechanical allodynia in mice. HMGB1-Ab or IgG at 1 mg/kg was administered i.p. 1 h before (**a**) or 8 days after i.p. administration of OHP at 5 mg/kg in mice. **c**, **d** The protein levels of HMGB1 in the plasma (**c**), dorsal root ganglion (**d**, left), sciatic nerve (**d**, middle), and hind paw (**d**, right) 5 h and day 3 and/or 8 after OHP treatment in mice. **e** Preventive effect of HMGB1-Ab on the OHP-induced mechanical hyperalgesia and allodynia in rats, as assessed by the paw pressure and von Frey tests, respectively. OHP at 5 mg/kg was administered i.p. every third day, four times in total, and HMGB1-Ab or non-immune IgG at 1 mg/kg was repeatedly administered i.p. 1 h before and 24 h after each dose of OHP, eight times in total, i.e., on days 0, 1, 3, 4, 6, 7, 9, and 10 of OHP treatment. **f** Plasma HMGB1 levels 5 h and day 3 or 12 after the onset of OHP treatment in rats. V, vehicle. Data show the mean with SEM for four (**a**), four to eight (**b**), five (**c**, left), eight to nine (**c**, right), and four to five (**d**) mice and for four to five (**e**), and five to six (**f**) rats. *P < 0.05, **P < 0.01 vs. vehicle in the vehicle-treated mice (**a**, **b**) or rats (**e**); ^†^*P* < 0.05, ^††^*P* < 0.01 vs. IgG in the OHP-treated mice (**a**, **b**) or rats (**e**)

We previously demonstrated that HMGB1 plays a critical role in the development and maintenance of peripheral neuropathy following repeated administration of paclitaxel or vincristine in rats and/or mice [4, 6]. Here, we tested whether HMGB1 was involved in oxaliplatin-induced neuropathy in mice and rats. In ddY mice, a single i.p. administration of oxaliplatin at 5 mg/kg developed prompt mechanical allodynia, as assessed by von Frey test, which lasted for 7 days or more (Fig. 1a). An anti-HMGB1-neutralizing antibody, preadministered i.p. at 1 mg/kg, completely prevented the development of oxaliplatin-induced mechanical allodynia in mice (Fig. 1a). The anti-HMGB1 antibody, when administered once at the same dose 8 days after oxaliplatin treatment, reversed the developed mechanical allodynia in mice (Fig. 1b). On the other hand, cold allodynia in mice was also detected 3 h after oxaliplatin treatment, but resistant to the anti-HMGB1-neutralizing antibody (Additional file 1: Figure S1A). Plasma HMGB1 levels in mice significantly increased 5 h and 8 days after oxaliplatin treatment (Fig. 1c). In contrast, no significant changes in the expression levels of HMGB1 in the DRG, sciatic nerves, and hind paws were detected 5 h and 3 and 8 days after oxaliplatin treatment in mice (Fig. 1d). Immunofluorescence analysis did not show remarkable alterations in the expression of HMGB1 in the DRG neurons 5 h and 8 days after oxaliplatin treatment in mice (Additional file 2: Figure S2). In Wistar rats, oxaliplatin, administered i.p. every third day, four times in total, slowly developed mechanical hyperalgesia and allodynia, as assessed by the paw pressure test and von Frey test, respectively (Fig. 1e), although a single i.p. administration of oxaliplatin did not cause hyperalgesia or allodynia in the rats in the preliminary experiments (data not shown). The anti-HMGB1-neutralizing antibody, administered i.p. at 1 mg/kg repeatedly on days 0, 1, 3, 4, 6, 7, 9, and 10 of oxaliplatin treatment, clearly prevented the development of oxaliplatin-induced hyperalgesia and allodynia (Fig. 1e), in agreement with the findings in the mouse model for oxaliplatin-induced peripheral neuropathy (see Fig. 1a). Interestingly, plasma HMGB1 levels in rats tended to increase 3 and 12 days, but not 5 h, after the onset of oxaliplatin treatment in rats (Fig. 1f), being greatly different from those in mice (see Fig. 1c).

**Fig. 2:**
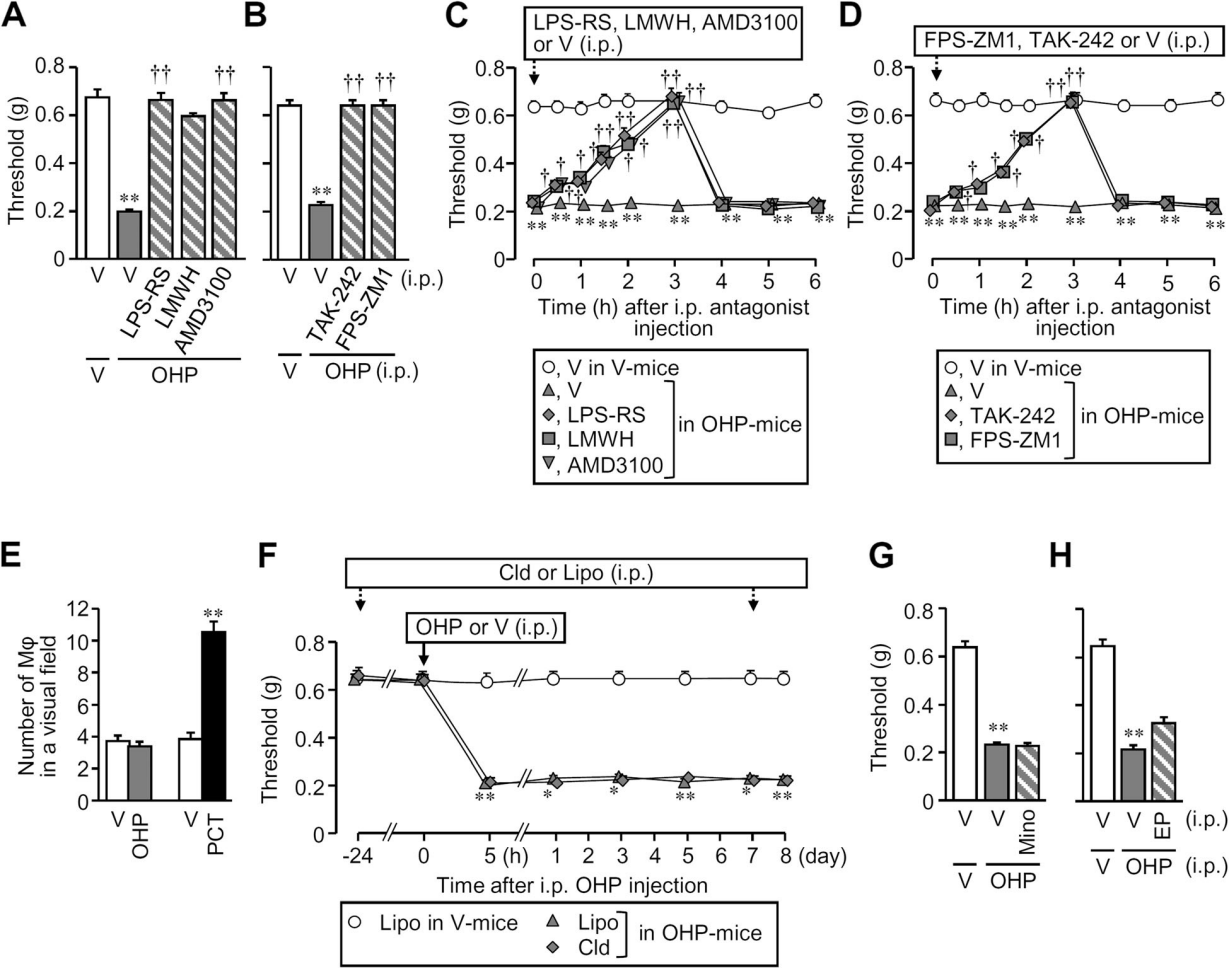
Roles of HMGB1-targeted receptors and macrophages (Mφ) in the oxaliplatin (OHP)-induced peripheral neuropathy in mice. **a**–**d** Preventive (**a**, **b**) and therapeutic (**c**, **d**) effects of distinct antagonists of TLR4, RAGE, and CXCR4 on the OHP-induced peripheral neuropathy in mice. The mice received i.p. administration of OHP at 5 mg/kg (**a**–**d**). LPS-RS at 0.5 mg/kg and TAK-242 at 3 mg/kg, TLR4 antagonists; low molecular weight heparin (LMWH) at 2.5 mg/kg and FPS-ZM1 at 1 mg/kg, which block RAGE; AMD3100 at 8 mg/kg, a CXCR4 antagonist or vehicle were administered i.p. 1 h before (**a**, **b**) or 8 days after (**c**, **d**) OHP treatment. The results show nociceptive thresholds assessed 7 days after OHP treatment (**a**, **b**) and the time course of nociceptive thresholds after i.p. administration of the antagonists (**c**, **d**). **e**–**h** Lack of involvement of Mφ in the OHP-induced peripheral neuropathy. **e** The number of Mφ in the sciatic nerve of the mice treated with OHP or paclitaxel (PCT). Mice received a single i.p. administration of OHP at 5 mg/kg or repeated i.p. administration of PCT at 4 mg/kg on days 0, 2, 4, and 6. The sciatic nerves were isolated from the mice on day 8 after administration of OHP and on day 9 of PCT treatment, and the number of Mφ was determined in a microscopic visual field. **f** Macrophage depletion does not affect the development of OHP-induced neuropathy in mice. Liposomal clodronate (Cld), a Mφ depletor, or the control liposome (Lipo) at 1.05 mg/mouse was injected i.p. to mice 24 h before or 7 days after i.p. OHP. The time course of nociceptive threshold before and after OHP treatment is shown in the mice treated with Cld or Lipo. **g**, **h** Lack of effect of minocycline (Mino), a microglia/Mφ inhibitor, or ethyl pyruvate (EP), known to inhibit HMGB1 release from Mφ, on the OHP-induced mechanical allodynia in mice. Mino at 30 mg/kg was administered i.p. 24 h and 1 h before and once a day for 6 days after OHP treatment, eight times in total (**g**), and EP at 80 mg/kg was administered i.p. 24 h and 1 h before and on days 1, 3, 5, and 7 after OHP treatment, six times in total (**h**). Nociceptive threshold determined 8 days after OHP treatment is shown (**g**, **h**). V, vehicle. Data show the mean with SEM for 4–5 (**a**, **f**–**h**), 5 (**b**–**d**), or 5–6 (**c**) mice and for 20 fields from 5 mice (**e**). *P < 0.05, **P < 0.01 vs. vehicle + vehicle (**a**–**e**, **g**, **h**) or Lipo in V mice (**f**); ^†^*P* < 0.05, ^††^*P* < 0.01 vs. vehicle + OHP (**a**–**d**)

We next asked whether well-known, pro-nociceptive receptors for HMGB1, such as TLR4, RAGE, and CXCR4 [7–10, 28], contributed to the oxaliplatin-induced peripheral neuropathy. A single i.p. preadministration of LPS-RS at 0.5 mg/kg and TAK-242 at 3 mg/kg, TLR4 antagonists; low molecular weight heparin (LMWH) at 2.5 mg/kg and FPS-ZM1 at 1 mg/kg, known to antagonize RAGE; or AMD3100 at 8 mg/kg, an CXCR4 antagonist, prevented the development of mechanical allodynia in mice treated with i.p. oxaliplatin at 5 mg/kg (Fig. 2a, b). Any of those receptor antagonists, when administered i.p. once 8 days after oxaliplatin treatment, reversed the mechanical allodynia in mice (Fig. 2c, d). Protein levels of RAGE in the DRG, but not the sciatic nerve, significantly increased 8 days after i.p. oxaliplatin at 5 mg/kg, while protein expression of TLR4 and CXCR4 in the DRG and sciatic nerve did not significantly change by oxaliplatin treatment (Additional file 3: Figure S3).

### Oxaliplatin-induced peripheral neuropathy in mice is largely independent of macrophages

Given our recent evidence for the critical role of macrophage-derived HMGB1 in paclitaxel-induced peripheral neuropathy [6], we asked whether oxaliplatin-induced neuropathy involved macrophages in mice. Strikingly, i.p. oxaliplatin at 5 mg/kg did not clearly cause macrophage infiltration or accumulation in the sciatic nerves, in contrast to the remarkable macrophage accumulation after paclitaxel treatment in mice (Additional file 4: Figure S4). The number of macrophages in the sciatic nerves did not change by oxaliplatin treatment, although it almost doubled by paclitaxel treatment (Fig. 2e). Treatment with liposomal clodronate, a macrophage depletor, dramatically decreased the number of macrophages in the spleen (Additional file 5: Figure S5A, B), whereas it neither prevented nor reversed the oxaliplatin-induced allodynia in mice (Fig. 2f). Likewise, repeated administration of minocycline at 30 mg/kg, a macrophage/microglia inhibitor, or ethyl pyruvate at 80 mg/kg, known to inhibit HMGB1 release from macrophages, did not block the oxaliplatin-induced mechanical allodynia (Fig. 2g, h). In cultured mouse macrophage-like RAW264.7 cells, paclitaxel at 0.1–1 μM significantly caused HMGB1 release, which was mimicked by oxaliplatin at 10 μM that was 100-fold higher than the effective concentration of paclitaxel (Additional file 6: Figure S6A). On the other hand, oxaliplatin even at 3 μM evoked HMGB1 release from the primary culture of rat Schwann cells (Additional file 6: Figure S6B). Thus, the HMGB1-dependent peripheral neuropathy caused by oxaliplatin appears largely independent of macrophages, which is quite different from the critical role of macrophages in a mouse model for paclitaxel-induced peripheral neuropathy [6, 31, 32].

### Thrombin-dependent prevention of oxaliplatin-induced peripheral neuropathy by TMα in mice

**Fig. 3:**
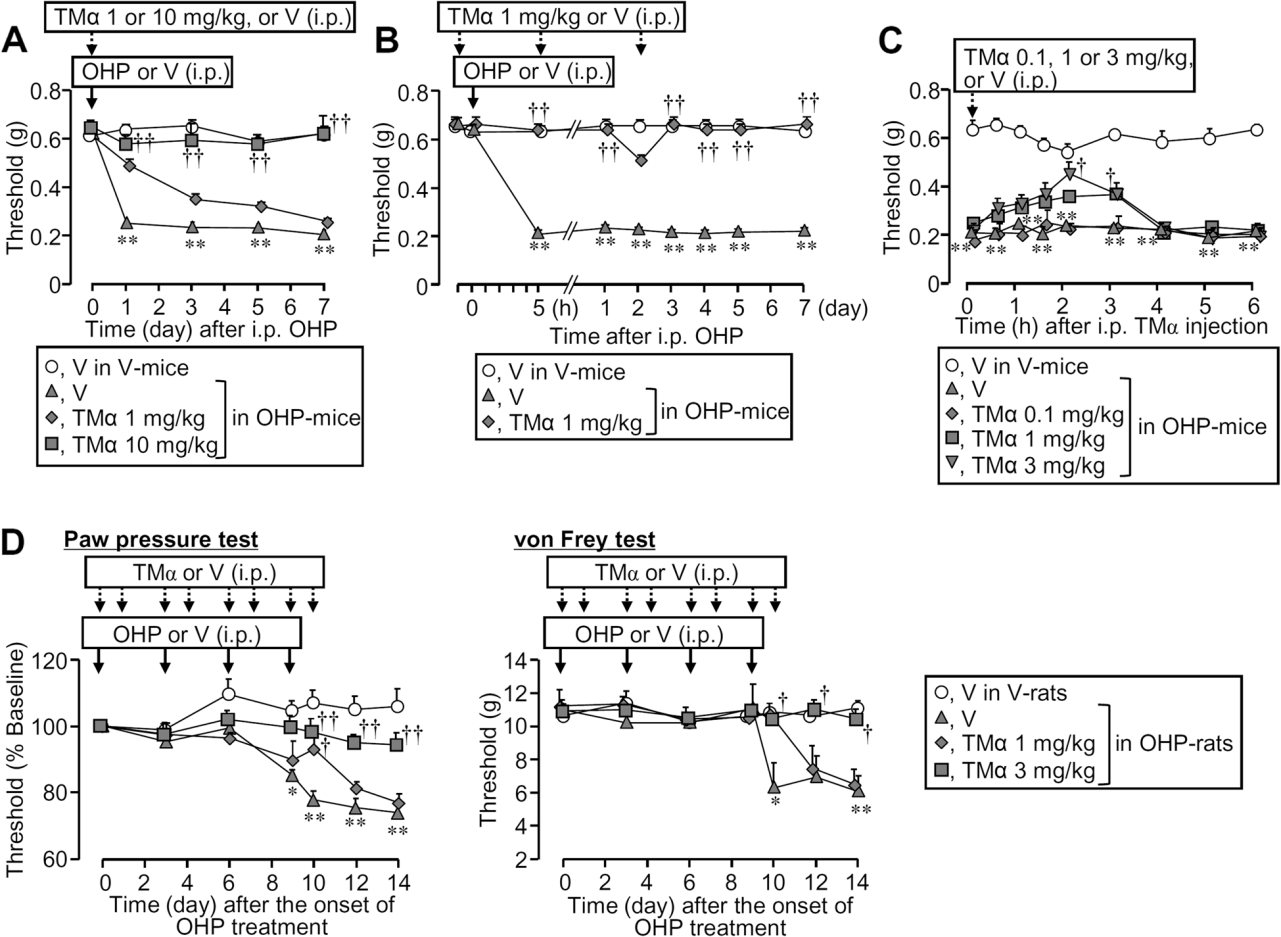
TMα abolishes oxaliplatin (OHP)-induced neuropathic allodynia in rodents. **a**–**c** Preventive (**a**, **b**) and therapeutic (**c**) effects of TMα on the peripheral neuropathy caused by i.p. administration of OHP at 5 mg/kg in mice. To evaluate the preventive effect, TMα at 1 or 10 mg/kg was administered i.p. once 1 h before OHP treatment (**a**) or three times 1 h before and 5 h and 2 days after OHP treatment (**b**). To examine the therapeutic effect, TMα at 0.1, 1, or 3 mg/kg was administered i.p. 8 days after OHP treatment (**c**). **d** Preventive effect of TMα on the mechanical hyperalgesia (paw pressure test) and allodynia (von Frey test) caused by four repeated i.p. administrations of OHP at 5 mg/kg on days 0, 3, 6, and 9 in rats. TMα at 1 or 3 mg/kg was administered i.p. on days 0, 1, 3, 4, 6, 7, 9, and 10 of OHP treatment. V, vehicle. Data show the mean with SEM for four to eight (**a**), five to six (**b**), and four to six (**c**) mice and for five rats (**d**). *P < 0.05, **P < 0.01 vs. vehicle + vehicle; ^†^*P* < 0.05, ^††^*P* < 0.01 vs. vehicle + OHP

**Fig. 4:**
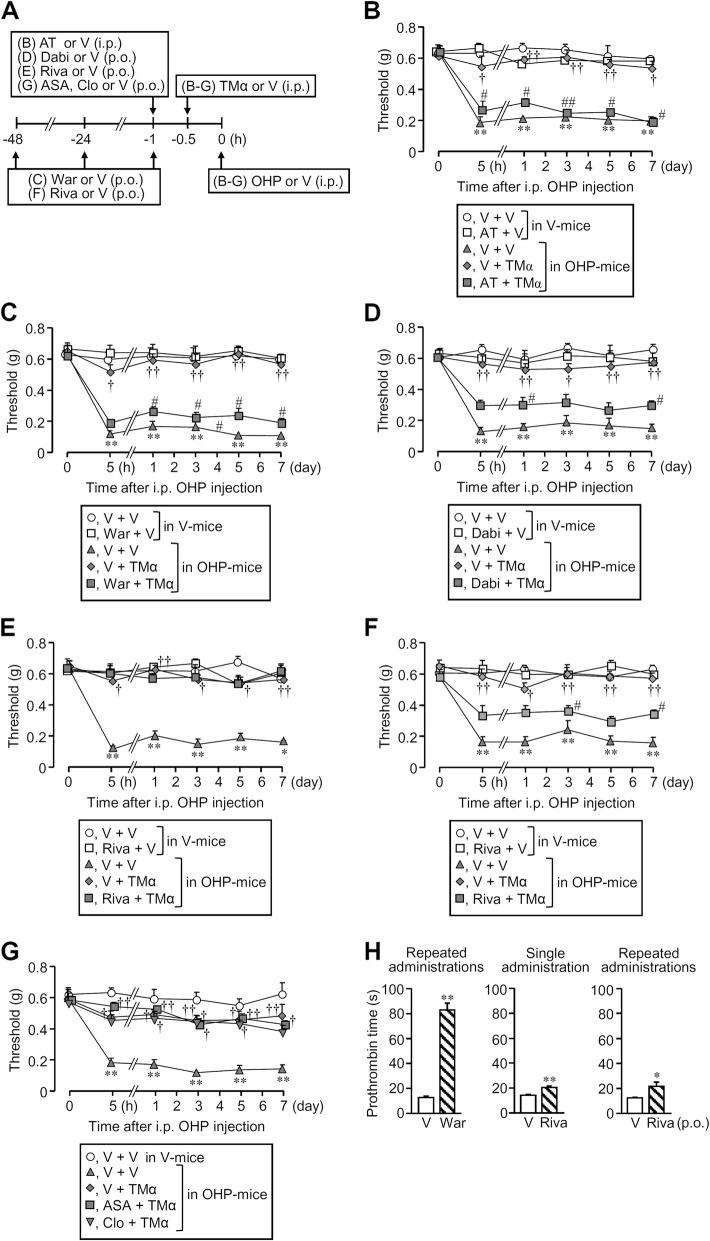
Anticoagulants, but not antiplatelet agents, cancel the preventive effect of TMα against oxaliplatin (OHP)-induced neuropathic allodynia in mice. **a** Drug administration schedule. TMα at 10 mg/kg was administered i.p. 0.5 h before i.p. OHP at 5 mg/kg (**b**–**g**). **b** Effect of argatroban (AT), a parenteral direct thrombin inhibitor, on the anti-neuropathic effect of TMα. AT at 10 mg/kg was administered i.p. 1 h before OHP treatment. **c** Effect of warfarin (War), a vitamin K antagonist, on the anti-neuropathic effect of TMα. War at 1 mg/kg was administered orally 48 h, 24 h, and 1 h before OHP treatment, three times in total. **d** Effect of dabigatran (Dabi), an oral direct thrombin inhibitor, on the anti-neuropathic effect of TMα. Dabi at 75 mg/kg was administered orally 1 h before OHP treatment. **e**, **f** Effect of rivaroxaban (Riva), an oral direct Xa inhibitor, on the anti-neuropathic effect of TMα. Riva at 10 mg/kg was administered orally once 1 h before OHP treatment (**e**) and repeatedly 48 h, 24 h, and 1 h before OHP treatment, three times in total (**f**). **g** Lack of effect of aspirin (ASA) and clopidogrel (Clo), antiplatelet agents, on the anti-neuropathic effect of TMα. ASA at 50 mg/kg or Clo at 10 mg/kg was administered orally once 1 h before OHP treatment. **h** Effects of War and Riva on the prothrombin time. Blood samples were collected 1 h after three repeated administrations of War and after single or three repeated administrations of Riva, as mentioned above (**a**). V, vehicle. Data show the mean with SEM for five mice. *P < 0.05, **P < 0.01 vs. vehicle + vehicle in vehicle-treated mice; ^†^*P* < 0.05, ^††^*P* < 0.01 vs. vehicle + vehicle in OHP-treated mice; ^#^*P* < 0.05, ^##^*P* < 0.01 vs. vehicle + TMα in OHP-treated mice

A single i.p. preadministration of TMα, capable of promoting thrombin-dependent degradation of HMGB1 [14–16], at 10 mg/kg prevented the development of mechanical allodynia caused by i.p. oxaliplatin at 5 mg/kg in mice (Fig. 3a). TMα at 1 mg/kg, when administered i.p. three times at 24-h intervals, mimicked the complete prevention of its single administration at 10 mg/kg (Fig. 3a, b). On the other hand, the acute cold allodynia caused by oxaliplatin treatment was resistant to TMα at 10 mg/kg (Additional file 1: Figure S1B), being consistent with the lack of effect of the anti-HMGB1-neutralizing antibody (Additional file 1: Figure S1A). TMα, when administered i.p. once at 0.1–3 mg/kg on day 8 after oxaliplatin treatment, reversed the continuing mechanical allodynia caused by oxaliplatin in a dose-dependent manner (Fig. 3c). Similarly, in the rat model for oxaliplatin-induced peripheral neuropathy, repeated i.p. administration of TMα at 1–3 mg/kg dose-dependently prevented the slowly developing mechanical hyperalgesia and allodynia following repeated i.p. administration of oxaliplatin at 5 mg/kg (Fig. 3d). Since thrombin is required for TMα-induced inhibition of the allodynia following intraplantar injection of HMGB1 in mice [15, 16], we next tested whether the preventive effect of TMα against the oxaliplatin-induced peripheral neuropathy involves endogenous thrombin. Argatroban, a direct thrombin inhibitor, preadministered i.p. at 10 mg/kg, abolished the preventive effect of TMα at 10 mg/kg against the development of neuropathic allodynia following i.p. oxaliplatin at 5 mg/kg in mice (Fig. 4a, b), indicating an essential role of endogenous thrombin in the effect of TMα on the oxaliplatin-induced peripheral neuropathy.

### The preventive effect of TMα against oxaliplatin-induced peripheral neuropathy is attenuated by clinically available oral anticoagulants, but not antiplatelet agents

A variety of oral anticoagulants used for the treatment of thrombotic diseases, which inhibit the enzymatic activity or generation of thrombin, might restrain the clinical usefulness of TMα in the prevention of CIPN. We thus tested if oral anticoagulants affected the TMα-induced prevention of oxaliplatin-induced peripheral neuropathy. As did a single parenteral administration of argatroban, a direct thrombin inhibitor (see Fig. 4a, b), three repeated daily oral administrations of warfarin at 1 mg/kg, a vitamin K antagonist, abolished the preventive effect of i.p. TMα at 10 mg/kg against the mechanical allodynia caused by i.p. oxaliplatin at 5 mg/kg (Fig. 4a, c). Similarly, a single oral administration of dabigatran at 75 mg/kg, an oral direct thrombin inhibitor, significantly attenuated the preventive effect of TMα against the oxaliplatin-induced mechanical allodynia (Fig. 4a, d). In contrast, a single oral administration of rivaroxaban at 10 mg/kg, a factor Xa inhibitor which belongs to direct oral anticoagulants (DOAC), failed to affect the TMα-induced prevention of the mechanical allodynia caused by oxaliplatin (Fig. 4a, e). However, three repeated oral administration of rivaroxaban at the same dose significantly reduced the preventive effect of TMα against the oxaliplatin-induced mechanical allodynia (Fig. 4a, f). In contrast, antiplatelet agents including aspirin and clopidogrel, administered orally at 50 mg/kg and 10 mg/kg, respectively, had no effect on nociceptive threshold by themselves (Additional file 7: Figure S7) and did not affect the preventive effect of TMα in the CIPN model (Fig. 4a, g). It was confirmed that repeated administration of warfarin greatly prolonged prothrombin time, while rivaroxaban, administered once or three times, caused relatively slight prolongation of prothrombin time (Fig. 4a, h).

### Repeated treatment with anticoagulants causes exacerbation of peripheral neuropathy and increase in plasma HMGB1 levels in mice treated with a subeffective dose of oxaliplatin

**Fig. 5:**
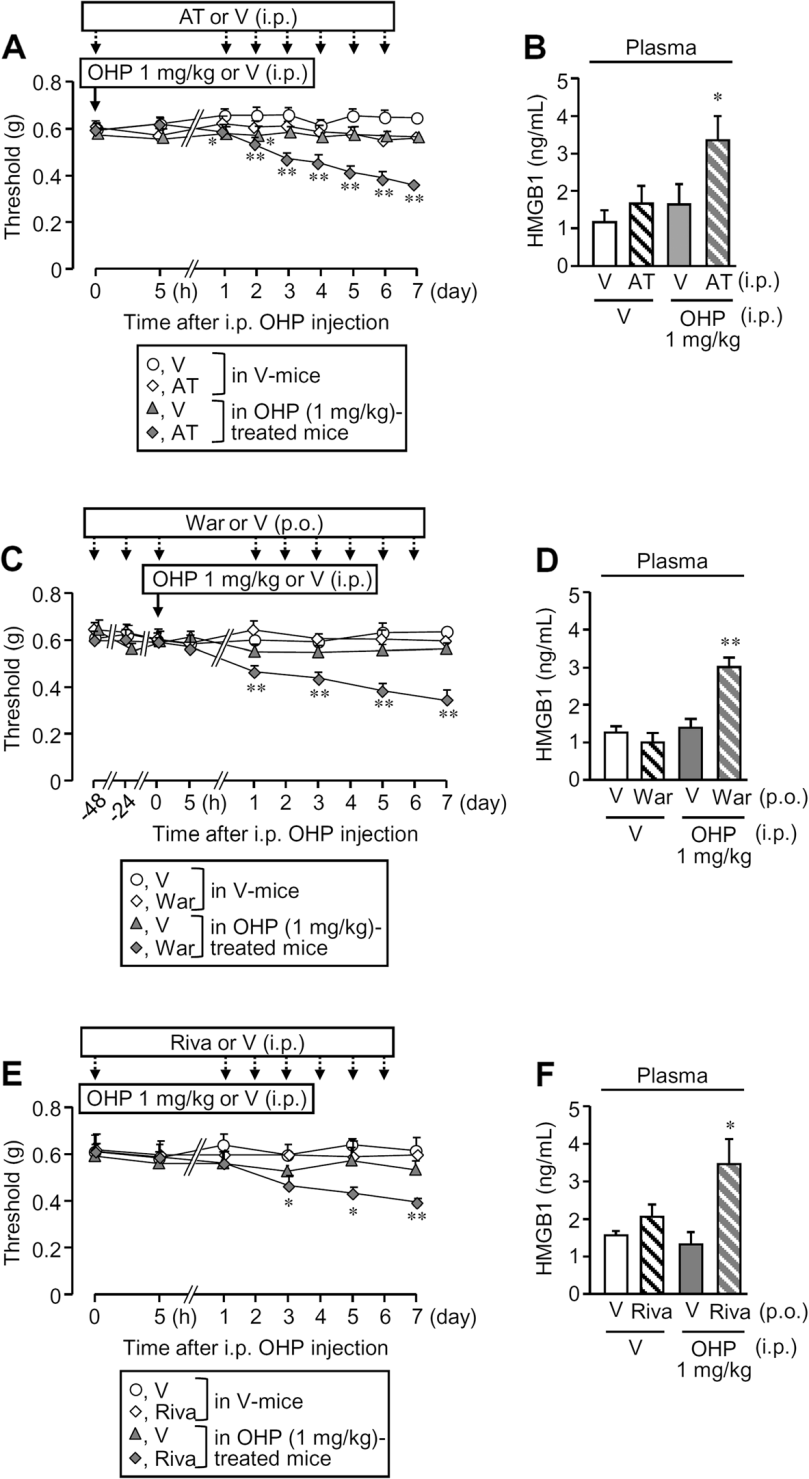
Long-term inhibition of enzymatic activity or generation of thrombin develops mechanical allodynia and elevates plasma HMGB1 levels in mice treated with oxaliplatin (OHP) at a subeffective dose. **a**, **b** Argatroban (AT), a parenteral direct thrombin inhibitor, at 10 mg/kg was administered i.p. 0.5 h before and once a day for 6 days after i.p. OHP at 1 mg/kg, a subeffective dose. **c**, **d** Warfarin (War), a vitamin K antagonist, at 1 mg/kg was administered orally 48 h, 24 h, and 0.5 h before and once a day for 6 days after treatment with the subeffective dose of OHP. **e**, **f** Rivaroxaban (Riva), an oral direct Xa inhibitor, at 10 mg/kg was administered orally 0.5 h before and once a day for 6 days after treatment with the subeffective dose of OHP. Nociceptive threshold was determined before and after OHP treatment (**a**, **c**, **e**), and blood samples for ELISA assay of plasma HMGB1 levels were obtained on day 7 after OHP treatment (**b**, **d**, **f**). V, vehicle. Data show the mean with SEM for six to eight (**a**) or five to eight (**b**) mice. *P < 0.05, **P < 0.01 vs. vehicle + vehicle

To test whether the endogenous TM/thrombin system functioned to degrade HMGB1 in the bloodstream and restrain the development of CIPN, we examined the effects of repeated administration of anticoagulants, which decrease the enzymatic activity or generation of thrombin, on the nociceptive threshold and plasma HMGB1 levels after oxaliplatin treatment in mice. Surprisingly, repeated administration of argatroban, warfarin, or rivaroxaban slowly developed mechanical allodynia in mice treated with i.p. oxaliplatin at a subeffective dose, 1 mg/kg (Fig. 5a, c, e), and significantly increased plasma HMGB1 levels on day 7 after the oxaliplatin treatment (Fig. 5b, d, f).

## Discussion

**Fig. 6:**
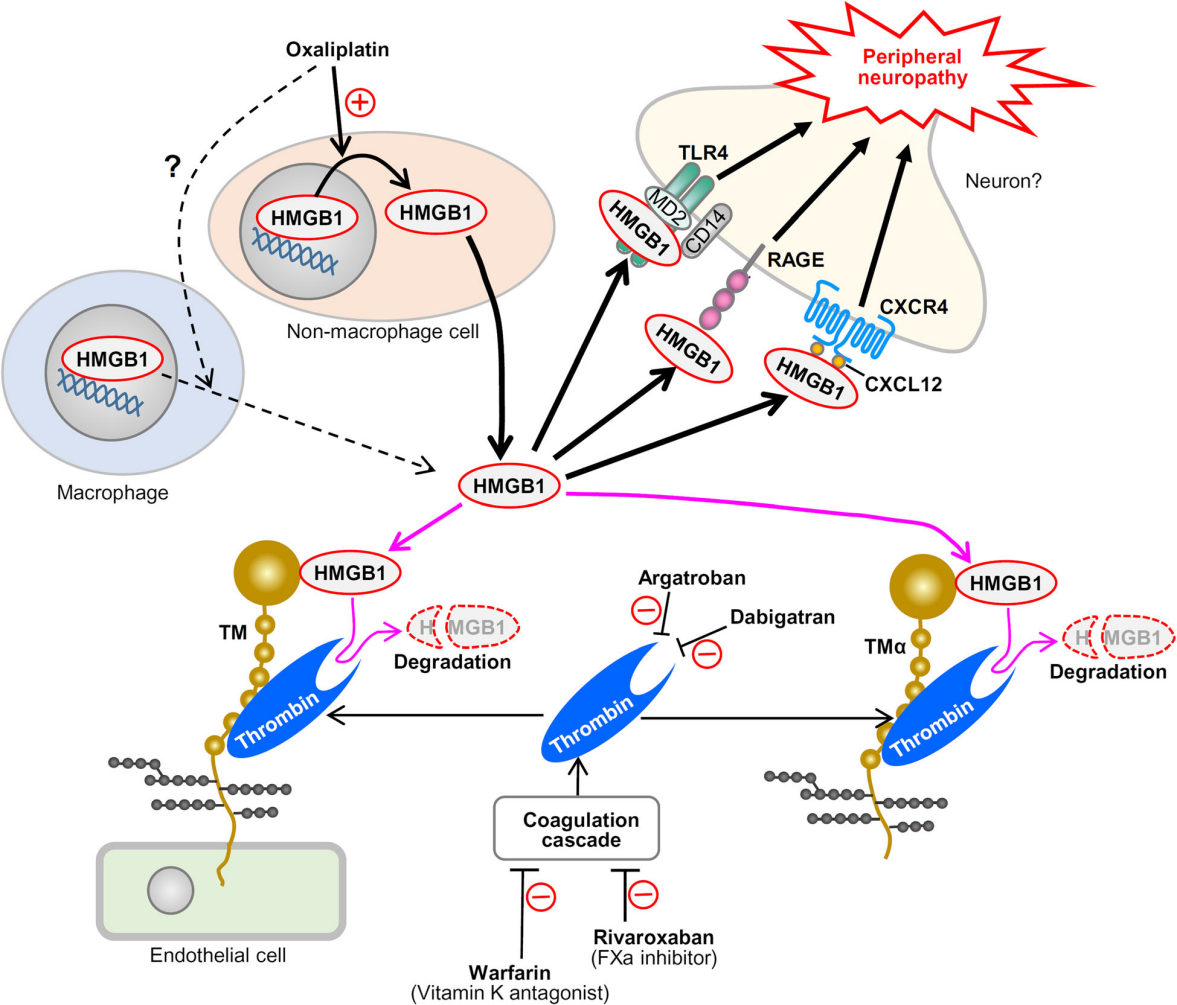
Hypothetical scheme for a causative role of HMGB1 in oxaliplatin-induced peripheral neuropathy and for thrombin-dependent anti-neuropathic effects of exogenously applied TMα and endogenous TM expressed in the endothelium. Oxaliplatin releases HMGB1 mainly from non-macrophage cells, which causes peripheral neuropathy via activation of TLR4, RAGE, and CXCL12/CXCR4 signaling, but not TLR5. Exogenously applied TMα and endothelial TM prevent oxaliplatin-induced neuropathy by promoting thrombin-dependent degradation of HMGB1. Anticoagulants, such as argatroban, dabigatran, warfarin, and rivaroxaban, cancel the anti-neuropathic activity of TMα and endothelial TM

The present study demonstrates the critical role of HMGB1 in the development and maintenance of oxaliplatin-induced peripheral neuropathy, which is mediated by RAGE, TLR4, and CXCR4. Most interestingly, our data suggest that the oxaliplatin-induced neuropathy is largely independent of macrophages, which is in contrast to the critical role of macrophages as the origin of HMGB1 involved in paclitaxel-induced neuropathy in our previous study [6]. Further, our study clearly demonstrates the crucial role of endogenous thrombin in the TMα-induced prevention of oxaliplatin-induced peripheral neuropathy and indicates that anticoagulants, which inhibit enzymatic activity or generation of thrombin, abolish or attenuate the preventive effects of TMα. Considering our findings that a long-term inhibition of thrombin or its generation caused neuropathic allodynia together with the elevation in plasma HMGB1 levels after treatment with a subeffective dose of oxaliplatin, we assume that the endogenous TM/thrombin system restrains the development of oxaliplatin-induced peripheral neuropathy by degrading HMGB1 released from unknown cells in response to oxaliplatin. Collectively, we propose that HMGB1, derived from non-macrophage cells, mediates oxaliplatin-induced peripheral neuropathy possibly through activation of TLR4, RAGE, and CXCL12/CXCR4 signaling, which is limited by the endogenous TM/thrombin system and attenuated by exogenously applied TMα in a thrombin-dependent manner (Fig. 6).

We have reported that HMGB1 is involved in the peripheral neuropathy caused by paclitaxel or vincristine in mice and/or rats [4, 6]. Given the present evidence for the involvement of HMGB1 in oxaliplatin-induced neuropathy, HMGB1 is considered a common key molecule in the pathogenesis of CIPN caused by distinct chemotherapeutics. The rapid increase in plasma HMGB1 levels (see Fig. 1c) supports the notion that extracellular HMGB1 are associated with the development of mechanical allodynia in mice, although the origins of plasma HMGB1 have yet to be identified. Nonetheless, it is to be noted that the acute cold allodynia caused by oxaliplatin in mice was resistant to the anti-HMGB1-neutralizing antibody or TMα (see Additional file 1: Figure S1), suggesting that the cold allodynia does not involve HMGB1, in agreement with the previous reports demonstrating that oxaliplatin and its metabolite, oxalate, cause ROS-mediated activation and prolyl hydroxylase inhibition-dependent sensitization of TRPA1, respectively, leading to cold hypersensitivity [33, 34]. The species differences in the development of oxaliplatin-induced mechanical allodynia between mice and rats might be associated with their distinct time course of the increases in plasma HMGB1 levels, since, apparently, plasma HMGB1 levels increased nearly in parallel with the development of mechanical allodynia/hyperalgesia in each of mice and rats (see Fig. 1c, f).

Surprisingly, macrophages, which are responsible as an origin of extracellular HMGB1 for the paclitaxel-induced peripheral neuropathy [6], do not appear to contribute to the neuropathy in the oxaliplatin-treated mice (see Fig. 2e–h). This is consistent with our findings that oxaliplatin was 10- to 100-fold less potent than paclitaxel in causing HMGB1 release from mouse macrophage-like RAW264.7 cells (see Additional file 6: Figure S6A). Cells that release HMGB1 in the oxaliplatin-treated mice have yet to be identified. Schwann cells in addition to neurons could be such candidates, since oxaliplatin released HMGB1 in a little lower concentration range in the primary rat Schwann cells than in RAW264.7 cells (see Additional file 6: Figure S6B). It has been reported that the mice with peripheral neuropathy caused by oxaliplatin and by paclitaxel have different changes in the peripheral immune system and nervous system [35]. For example, the mice with oxaliplatin-induced neuropathy have increased interleukin-4-positive lymphocytes in the spleen and decreased regulatory T cells in the lymph node, whereas no such phenomena are detectable in the mice with paclitaxel-induced neuropathy. On the other hand, paclitaxel, but not oxaliplatin, causes expression of ATF-3, a neuropathy marker, in IB4-positive and NF200-positive nerves in the DRG and also astrocyte activation and increased protein levels of inflammatory cytokines and chemokines in the spinal cord. There is also evidence that oxaliplatin and paclitaxel impair Schwann cells via distinct mechanisms, i.e., the former causes mitochondrial dysfunction in Schwann cells, while the latter leads to dedifferentiation of Schwann cells into an immature state [36]. Thus, paclitaxel and oxaliplatin have greatly different effects on the immune system, neurons, and Schwann cells, which might be associated with the present findings that macrophages and non-macrophage cells were responsible as cellular origins of HMGB1 in the mice with the neuropathic pain caused by paclitaxel and oxaliplatin, respectively.

It is particularly of interest, in terms of clinical application, that TMα almost completely prevented the development of painful neuropathy following a single dose of oxaliplatin in mice or repeated doses of oxaliplatin in rats (see Fig. 3), in agreement with our previous reports showing the preventive effects of TMα against CIPN caused by paclitaxel or vincristine in rats and/or mice [4, 6]. It is also noteworthy that post-treatment with TMα reversed the established allodynia following oxaliplatin treatment, as reported in the paclitaxel-induced neuropathy model in rats [4]. Recent advance in cancer chemotherapy as well as molecular-targeted agents has dramatically improved the prognosis of cancer patients, whereas many of severe side effects of chemotherapeutics including CIPN, which terribly impair patients’ quality of life or interrupt the continuation of cancer therapy, remain to be resolved. Given the absence of an effective pharmacotherapy to prevent or treat CIPN [37], our present and previous preclinical studies [4, 6] showing the effectiveness of TMα in preventing CIPN may unveil a novel strategy to lessen a burden in cancer patients undergoing chemotherapy.

There is in vitro evidence that TMα consisting of TM’s D1–D3, produced by the Chinese hamster ovary (CHO) cell engineering system, sequesters HMGB1 with D1 and then promotes its degradation by thrombin binding to D2 [14–16]. Although TM’s D1 alone appears to exhibit anti-inflammatory activity [38, 39], our in vivo studies employing mice have clearly demonstrated that TMα-induced prevention of the allodynia following intraplantar injection of HMGB1 requires endogenous thrombin [16] and that yeast-generated TM’s D1–D3, but not D1 or D2 alone, mimics the preventive effect of the CHO-generated TMα against HMGB1-induced allodynia [15]. Therefore, it is quite understandable that the preventive effect of TMα against the oxaliplatin-induced neuropathic allodynia was abolished or attenuated by systemic administration of argatroban and dabigatran, parenteral and oral direct thrombin inhibitors, respectively, in the present study (see Fig. 4). Our findings that warfarin, the vitamin K antagonist, and rivaroxaban, the factor Xa inhibitor, capable of reducing thrombin generation, suppressed the TMα-induced prevention of CIPN (see Fig. 4) caution the possibility that TMα may not be effective as a prophylactic agent for CIPN in cancer patients undergoing anticoagulant therapy for thrombotic diseases, unless the use of anticoagulants is temporarily stopped. However, it is noteworthy that TMα itself exhibits anticoagulant activity by activating protein C [14], which might compensate for the suspension of the use of other anticoagulants and might even be reasonable to avoid hemorrhage, and that antiplatelet agents never affect the preventive effect of TMα against CIPN (see Fig. 4g).

It is intriguing and of critical importance from a clinical perspective that daily treatment with argatroban, warfarin, or rivaroxaban gradually developed mechanical allodynia in mice receiving i.p. oxaliplatin at 1 mg/kg, a subeffective dose (see Fig. 5), suggesting that the severity of CIPN might be correlated with anticoagulant therapy and that endogenous thrombin has anti-nociceptive or anti-neuropathic property. Our retrospective cohort study to detect the effects of anticoagulants on the severity of CIPN is now in progress, because there is no such clinical evidence in the literature information, to our knowledge. Further, our findings that the inhibition of activity or generation of thrombin elevated plasma HMGB1 levels in the mice treated with the subeffective dose of oxaliplatin (see Fig. 5) suggest that the endothelial TM-dependent degradation of HMGB1 by endogenous thrombin potentially protects against the development of CIPN (Fig. 6). The endogenous thrombin-dependent suppression of CIPN could also involve TM expressed in monocytes, which plays a role in the protection against of DIC [40].

## Conclusions

In conclusion, oxaliplatin-induced peripheral neuropathy appears to involve the activation of TLR4, RAGE, and CXCR4, by HMGB1 derived from non-macrophage cells, and TMα suppresses oxaliplatin-induced neuropathy in a thrombin-dependent manner, an effect canceled by anticoagulants that inhibit the generation of thrombin. Further, our study strongly suggests that the endothelial TM/thrombin system potentially functions to protect against the development of CIPN following oxaliplatin treatment.

## Supplementary information

**Additional file 1: Figure S1.** Effect of an anti-HMGB1-neutralizing antibody (HMGB1-Ab) and TMα on the oxaliplatin (OHP)-induced cold allodynia in mice. HMGB1-Ab at 1 mg/kg or IgG at 1 mg/kg (A) and TMα at 10 mg/kg (B) or vehicle (V) were administered i.p. 1 h before i.p. OHP at 5 mg/kg. Cold allodynia was measured 3 h after i.p. OHP. Data show the mean with S.E.M for 5-7 mice. ^*^P<0.05 vs. vehicle + vehicle.

**Additional file 2: Figure S2.** Immunofluorescence staining of HMGB1 in the DRG at the L5 spinal level 5 h (A) or 8 days (B) after oxaliplatin (OHP) treatment in mice. Mice received i.p. administration of OHP at 5 mg/kg or vehicle (V). Nuclei were stained with H33342 (blue), and HMGB1 were stained with an anti-HMGB1 chicken polyclonal antibody (red). Control, chicken IgG; Scale bar, 50 μm.

**Additional file 3: Figure S3.** Protein levels of TLR4, RAGE and CXCR4 in the DRG and sciatic nerve after oxaliplatin (OHP) treatment in mice. Typical photographs of Western blotting and quantified data by densitometry are shown. The mice received i.p. administration of OHP at 5 mg/kg. The DRG and sciatic nerve were excised 8 days after OHP treatment. Data show the mean with S.E.M for 7-8 mice. ^*^P<0.05 vs. vehicle.

**Additional file 4: Figure S4.** Immunofluorescence staining of F4/80, a macrophage marker, in the sciatic nerves excised from the mice with peripheral neuropathy caused by oxaliplatin (OHP) or paclitaxel (PCT). Mice received a single i.p. administration of OHP at 5 mg/kg, or repeated i.p. administration of paclitaxel at 4 mg/kg on days 0, 2, 4 and 6. The sciatic nerves were isolated from the mice on day 8 after administration of OHP and on day 9 of PCT treatment. Red, staining of F4/80 (macrophage); blue, DAPI (nucleus). Scale bar, 50 μm. Arrows indicate macrophages.

**Additional file 5: Figure S5.** Confirmation of liposomal clodronate-induced macrophage depletion in the isolated spleen. Liposomal clodronate (Cld), a macrophage depletor, or the control liposome (Lipo) at 1.05 mg/mouse was injected i.p. to mice 24 h before and 7 days after i.p. oxaliplatin (OHP) 5 mg/kg. On day 8 after OHP treatment, F4/80^+^ / CD11b^+^ macrophages in the isolated spleen was detected (A) and counted (B) by flowcytometry. Data show the mean with S.E.M. for 4-5 mice (B). **P<0.01 vs. vehicle + Lipo (B).

**Additional file 6: Figure S6.** Oxaliplatin (OHP)-induced HMGB1 release from macrophage-like RAW264.7 cells or the primary culture of rat Schwann cells. RAW264.7 cells were stimulated with OHP or paclitaxel (PCT) for 24 h (A), and Schwann cells were stimulated with OHP for 48 h (B). The protein levels of HMGB1 released into the culture medium were determined by ELISA. Data show the mean with S.E.M for 5 (A) or 6 (B) different experiments. ^**^P<0.01 vs. vehicle.

**Additional file 7: Figure S7.** Effect of antiplatelet drugs on nociceptive threshold. (A) The drug administration schedules. (B) Vehicle (V) for TMα was administered i.p. 0.5 h before i.p. V for oxaliplatin (OHP). Asprin (Asp) at 50 mg/kg, clopidogrel (Clo) at 10 mg/kg or V was administered p.o. 1 h before i.p. V for oxaliplatin. Data show the mean with S.E.M for 5 mice.

## Data Availability

All data generated or analyzed during this study are included in this published article [and its supplementary information file].

## Abbreviations

CIPN: Chemotherapy-induced peripheral neuropathy
DAMP: Damage-associated molecular pattern
DAPI: 4,6-Diamidino-2-phenylindole dihydrochloride
DOAC: Direct oral anticoagulants
DRG: Dorsal root ganglia
GAPDH: Glyceraldehyde 3-phosphate dehydrogenase
HMGB1: High mobility group box 1
LMWH: Low molecular weight heparin
LPS-RS: Lipopolysaccharide from *Rhodobacter sphaeroides*
RAGE: Receptor for advance glycation end products
TLR: Toll-like receptor
TM: Thrombomodulin
