# Supplementary material for: Role of non-macrophage cell-derived HMGB1 in oxaliplatin-induced peripheral neuropathy and its prevention by the thrombin/thrombomodulin system in rodents: negative impact of anticoagulants
Source: J Neuroinflammation. 2019 Oct 30;16:199. doi: 10.1186/s12974-019-1581-6 (PMC6822350; doi:10.1186/s12974-019-1581-6)
Supplement: Supplementary file 1 — Additional file 1: Figure S1. Effect of an anti-HMGB1-neutralizing antibody (HMGB1-Ab) and TMα on the oxaliplatin (OHP)-induced cold allodynia in mice. HMGB1-Ab at 1 mg/kg or IgG at 1 mg/kg (A) and TMα at 10 mg/kg (B) or vehicle (V) were administered i.p. 1 h before i.p. OHP at 5 mg/kg. Cold allodynia was measured 3 h after i.p. OHP. Data show the mean with S.E.M for 5-7 mice. *P<0.05 vs. vehicle + vehicle. [file 12974_2019_1581_MOESM1_ESM.pdf]

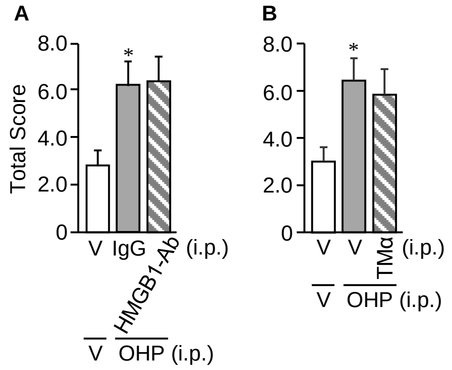

Additional file 1: Figure S1. Effect of an anti-HMGB1-neutralizing antibody (HMGB1-Ab) and TMα on the oxaliplatin (OHP)-induced cold allodynia in mice.
HMGB1-Ab at 1 mg/kg or IgG at 1 mg/kg (A) and TMα at 10 mg/kg (B) or vehicle (V) were administered i.p. 1 h before i.p. OHP at 5 mg/kg. Cold allodynia was measured 3 h after i.p. OHP. Data show the mean with S.E.M for 5-7 mice. *P<0.05 vs. vehicle + vehicle.
