# Supplementary material for: Role of non-macrophage cell-derived HMGB1 in oxaliplatin-induced peripheral neuropathy and its prevention by the thrombin/thrombomodulin system in rodents: negative impact of anticoagulants
Source: J Neuroinflammation. 2019 Oct 30;16:199. doi: 10.1186/s12974-019-1581-6 (PMC6822350; doi:10.1186/s12974-019-1581-6)
Supplement: Supplementary file 2 — Additional file 2: Figure S2. Immunofluorescence staining of HMGB1 in the DRG at the L5 spinal level 5 h (A) or 8 days (B) after oxaliplatin (OHP) treatment in mice. Mice received i.p. administration of OHP at 5 mg/kg or vehicle (V). Nuclei were stained with H33342 (blue), and HMGB1 were stained with an anti-HMGB1 chicken polyclonal antibody (red). Control, chicken IgG; Scale bar, 50 μm. [file 12974_2019_1581_MOESM2_ESM.pdf]

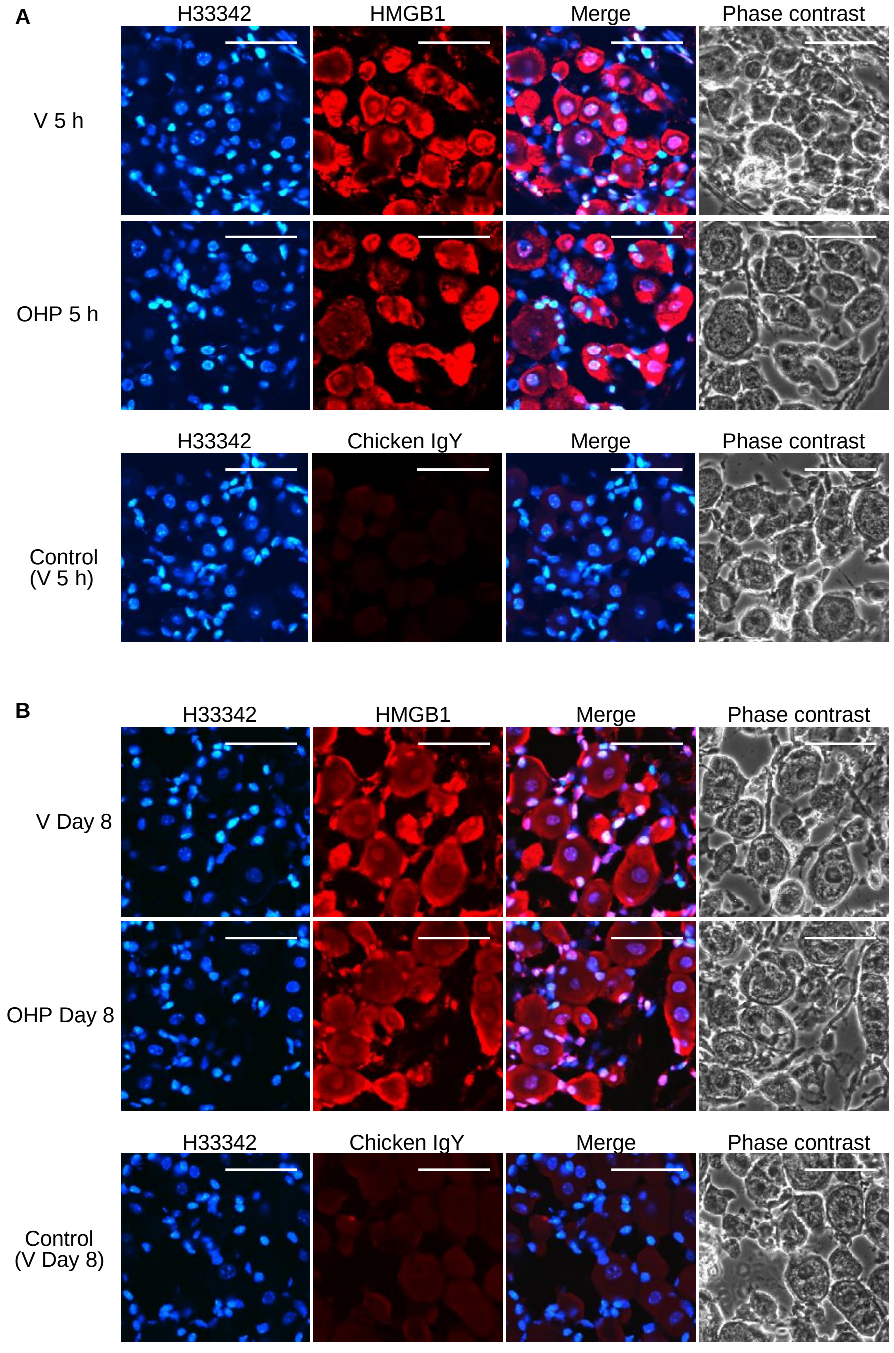

Additional file 2: Figure S2. Immunofluorescence staining of HMGB1 in the DRG at the L5 spinal level 5 h (A) or 8 days (B) after oxaliplatin (OHP) treatment in mice. Mice received i.p. administration of OHP at 5 mg/kg or vehicle (V). Nuclei were stained with H33342 (blue), and HMGB1 were stained with an anti-HMGB1 chicken polyclonal antibody (red). Control, chicken IgY; Scale bar, 50 µm.
