# Supplementary material for: Role of non-macrophage cell-derived HMGB1 in oxaliplatin-induced peripheral neuropathy and its prevention by the thrombin/thrombomodulin system in rodents: negative impact of anticoagulants
Source: J Neuroinflammation. 2019 Oct 30;16:199. doi: 10.1186/s12974-019-1581-6 (PMC6822350; doi:10.1186/s12974-019-1581-6)
Supplement: Supplementary file 3 — Additional file 3: Figure S3. Protein levels of TLR4, RAGE and CXCR4 in the DRG and sciatic nerve after oxaliplatin (OHP) treatment in mice. Typical photographs of Western blotting and quantified data by densitometry are shown. The mice received i.p. administration of OHP at 5 mg/kg. The DRG and sciatic nerve were excised 8 days after OHP treatment. Data show the mean with S.E.M for 7-8 mice. *P<0.05 vs. vehicle. [file 12974_2019_1581_MOESM3_ESM.pdf]

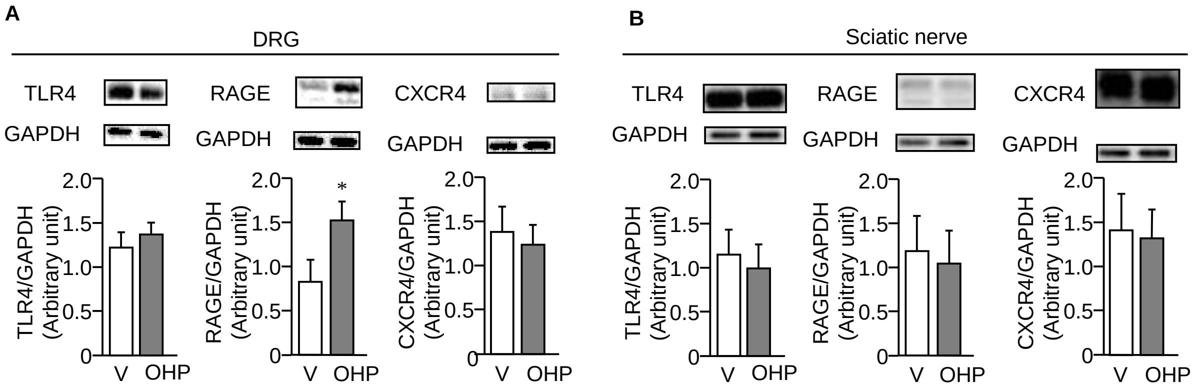

Additional file 3: Figure S3. Protein levels of TLR4, RAGE and CXCR4 in the DRG and sciatic nerve after oxaliplatin (OHP) treatment in mice. Typical photographs of Western blotting and quantified data by densitometry are shown. The mice received i.p. administration of OHP at 5 mg/kg. The DRG and sciatic nerve were excised 8 days after OHP treatment. Data show the mean with S.E.M for 7-8 mice. *P<0.05 vs. vehicle.
