# Supplementary material for: Role of non-macrophage cell-derived HMGB1 in oxaliplatin-induced peripheral neuropathy and its prevention by the thrombin/thrombomodulin system in rodents: negative impact of anticoagulants
Source: J Neuroinflammation. 2019 Oct 30;16:199. doi: 10.1186/s12974-019-1581-6 (PMC6822350; doi:10.1186/s12974-019-1581-6)
Supplement: Supplementary file 4 — Additional file 4: Figure S4. Immunofluorescence staining of F4/80, a macrophage marker, in the sciatic nerves excised from the mice with peripheral neuropathy caused by oxaliplatin (OHP) or paclitaxel (PCT). Mice received a single i.p. administration of OHP at 5 mg/kg, or repeated i.p. administration of paclitaxel at 4 mg/kg on days 0, 2, 4 and 6. The sciatic nerves were isolated from the mice on day 8 after administration of OHP and on day 9 of PCT treatment. Red, staining of F4/80 (macrophage); blue, DAPI (nucleus). Scale bar, 50 μm. Arrows indicate macrophages. [file 12974_2019_1581_MOESM4_ESM.pdf]

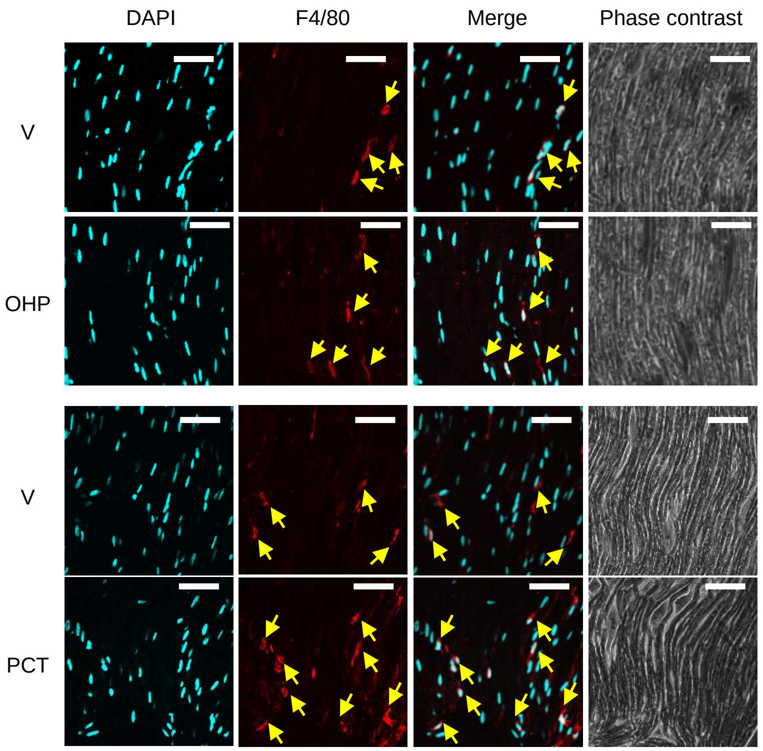

Additional file 4: Figure S4. Immunofluorescence staining of F4/80, a macrophage marker, in the sciatic nerves excised from the mice with peripheral neuropathy caused by oxaliplatin (OHP) or paclitaxel (PCT). Mice received a single i.p. administration of OHP at 5 mg/kg, or repeated i.p. administration of paclitaxel at 4 mg/kg on days 0, 2, 4 and 6. The sciatic nerves were isolated from the mice on day 8 after administration of OHP and on day 9 after the onset of PCT treatment. Red, staining of F4/80 (macrophage); blue, DAPI (nucleus). Scale bar, 50 µm. Arrows indicate macrophages.
