# Supplementary material for: Role of non-macrophage cell-derived HMGB1 in oxaliplatin-induced peripheral neuropathy and its prevention by the thrombin/thrombomodulin system in rodents: negative impact of anticoagulants
Source: J Neuroinflammation. 2019 Oct 30;16:199. doi: 10.1186/s12974-019-1581-6 (PMC6822350; doi:10.1186/s12974-019-1581-6)
Supplement: Supplementary file 5 — Additional file 5: Figure S5. Confirmation of liposomal clodronate-induced macrophage depletion in the isolated spleen. Liposomal clodronate (Cld), a macrophage depletor, or the control liposome (Lipo) at 1.05 mg/mouse was injected i.p. to mice 24 h before and 7 days after i.p. oxaliplatin (OHP) 5 mg/kg. On day 8 after OHP treatment, F4/80+ / CD11b+ macrophages in the isolated spleen was detected (A) and counted (B) by flowcytometry. Data show the mean with S.E.M. for 4-5 mice (B). **P<0.01 vs. vehicle + Lipo (B). [file 12974_2019_1581_MOESM5_ESM.pdf]

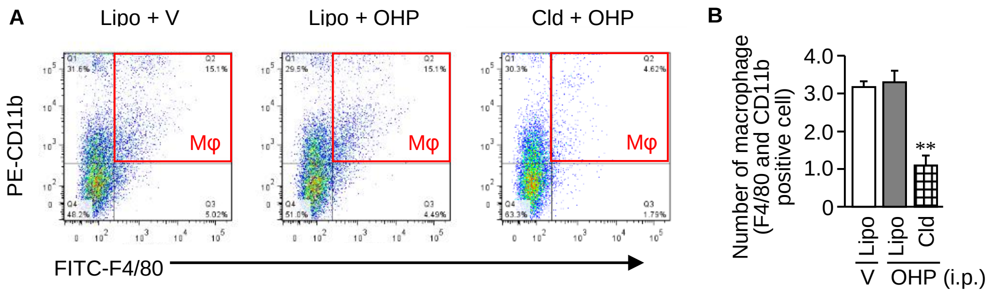

Additional file 5: Figure S5. Confirmation of liposomal clodronate-induced macrophage depletion in the isolated spleen. Liposomal clodronate (Cld), a macrophage depletor, or the control liposome (Lipo) at 1.05 mg/mouse was injected i.p. to mice 24 h before and 7 days after i.p. oxaliplatin (OHP) 5 mg/kg. On day 8 after OHP treatment, $F4/80^+$ / $CD11b^+$ macrophages in the isolated spleen was detected (A) and counted (B) by flowcytometry. Data show the mean with S.E.M. for 4-5 mice (B). **P<0.01 vs. vehicle + Lipo (B).
