# Supplementary material for: Role of non-macrophage cell-derived HMGB1 in oxaliplatin-induced peripheral neuropathy and its prevention by the thrombin/thrombomodulin system in rodents: negative impact of anticoagulants
Source: J Neuroinflammation. 2019 Oct 30;16:199. doi: 10.1186/s12974-019-1581-6 (PMC6822350; doi:10.1186/s12974-019-1581-6)
Supplement: Supplementary file 6 — Additional file 6: Figure S6. Oxaliplatin (OHP)-induced HMGB1 release from macrophage-like RAW264.7 cells or the primary culture of rat Schwann cells. RAW264.7 cells were stimulated with OHP or paclitaxel (PCT) for 24 h (A), and Schwann cells were stimulated with OHP for 48 h (B). The protein levels of HMGB1 released into the culture medium were determined by ELISA. Data show the mean with S.E.M for 5 (A) or 6 (B) different experiments. **P<0.01 vs. vehicle. [file 12974_2019_1581_MOESM6_ESM.pdf]

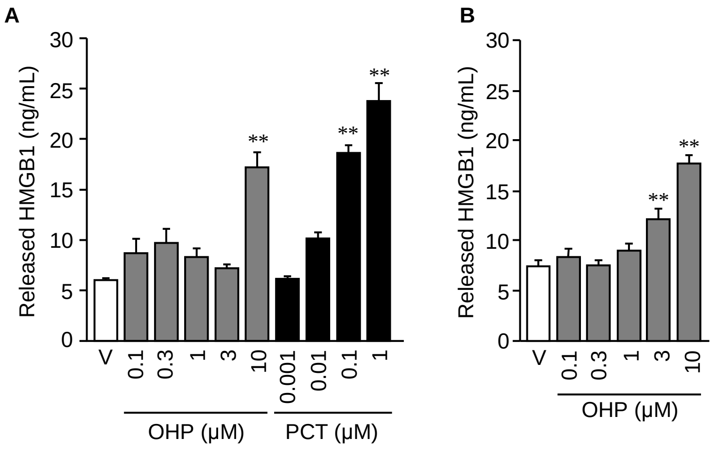

Additional file 6: Figure S6. Oxaliplatin (OHP)-induced HMGB1 release from macrophage-like RAW264.7 cells or the primary culture of rat Schwann cells. RAW264.7 cells were stimulated with OHP or paclitaxel (PCT) for 24 h (A), and Schwann cells were stimulated with OHP for 48 h (B). The protein levels of HMGB1 released into the culture medium were determined by ELISA. Data show the mean with S.E.M for 5 (A) or 6 (B) different experiments. **$P<0.01$ vs. vehicle.
