# Supplementary material for: Role of non-macrophage cell-derived HMGB1 in oxaliplatin-induced peripheral neuropathy and its prevention by the thrombin/thrombomodulin system in rodents: negative impact of anticoagulants
Source: J Neuroinflammation. 2019 Oct 30;16:199. doi: 10.1186/s12974-019-1581-6 (PMC6822350; doi:10.1186/s12974-019-1581-6)
Supplement: Supplementary file 7 — Additional file 7: Figure S7. Effect of antiplatelet drugs on nociceptive threshold. (A) The drug administration schedules. (B) Vehicle (V) for TMα was administered i.p. 0.5 h before i.p. V for oxaliplatin (OHP). Asprin (Asp) at 50 mg/kg, clopidogrel (Clo) at 10 mg/kg or V was administered p.o. 1 h before i.p. V for oxaliplatin. Data show the mean with S.E.M for 5 mice. [file 12974_2019_1581_MOESM7_ESM.pdf]

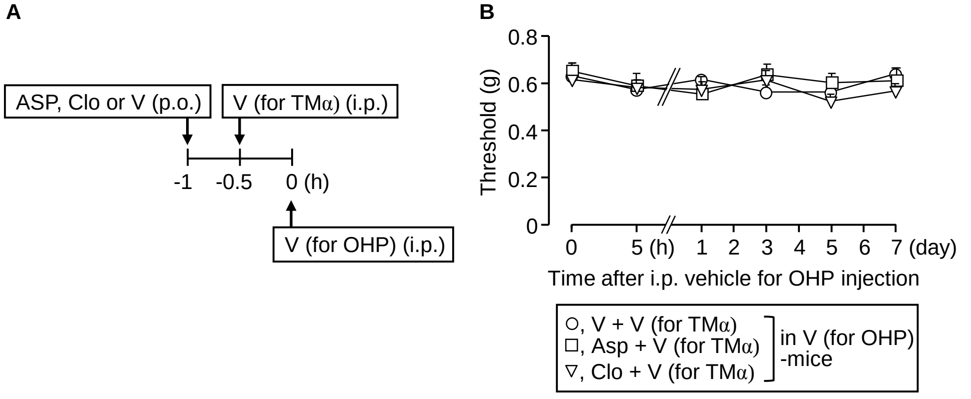

Additional file 7: Figure S7. Effect of antiplatelet drugs on nociceptive threshold. (A) The drug administration schedules. (B) Vehicle (V) for TMα was administered i.p. 0.5 h before i.p. V for oxaliplatin (OHP). Asprin (Asp) at 50 mg/kg, clopidogrel (Clo) at 10 mg/kg or V was administered p.o. 1 h before i.p. V for oxaliplatin. Data show the mean with S.E.M for 5 mice.
